# Alpha and beta diversity jointly drive the aboveground biomass in temperate and tropical forests

**DOI:** 10.1002/ece3.10487

**Published:** 2023-08-31

**Authors:** Jie Yao, Jihong Huang, Runguo Zang

**Affiliations:** ^1^ Ecology and Nature Conservation Institute, Chinese Academy of Forestry Key Laboratory of Forest Ecology and Environment of National Forestry and Grassland Administration Beijing China; ^2^ Co‐Innovation Center for Sustainable Forestry in Southern China Nanjing Forestry University Nanjing China

**Keywords:** aboveground biomass, biodiversity–ecosystem functioning, functional diversity, spatial scales, species diversity, α‐ and β diversity

## Abstract

Changes in biodiversity often affect ecosystem functioning. However, most previous biodiversity and ecosystem functioning (BEF) studies have generally been limited to very small spatial grains. Thus, knowledge regarding the biodiversity–ecosystem functioning relationships across spatial scales is lacking. Moreover, the multiscale nature of biodiversity, and specifically β diversity (i.e., spatial heterogeneity in species composition) was still largely missing in BEF studies. Here, using the vegetation and functional trait data collected from four 6‐ha forest dynamics plots (FDPs) in temperate and tropical forests in China, we examine the scale‐dependent relationships between tree diversity and the aboveground biomass (AGB), as well as the roles of species spatial heterogeneity in determining the AGB. In tropical forests, the effect of species richness on AGB decreased with spatial grains, while functional dominance played a stronger role at larger spatial grains. In temperate forests, positive relationship between diversity and AGB occurred at all spatial grains, especially on smaller scales. In both temperate and tropical forests, β diversity was positively correlated with AGB, but weaker than α diversity in determining AGB. Overall, complementarity and selection hypothesis play dominant role in determining AGB in temperate and tropical forests, respectively. The roles of these underlying mechanisms are more pronounced with increasing spatial scales. β diversity, a hitherto underexplored facet of biodiversity, is likely to increase ecosystem functions by species spatial turnover and should not be neglected in BEF explorations. Our findings have practical implications for forest management and demonstrate that biotic heterogeneity plays an important positive role in ecosystem functioning.

## Graphical abstract

Roles of α‐ and β‐diversity in determining AGB were examined. The roles of complementarity and selection are scale‐dependent. β diversity is likely to increase ecosystem functions via species spatial turnover.

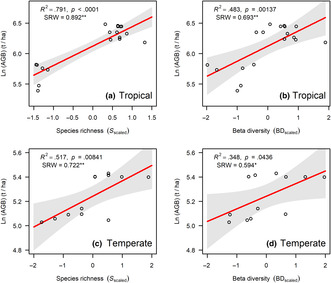

## 1 INTRODUCTION

The important roles of biodiversity in maintaining ecosystem functions (Cardinale et al., 2006, 2012; Hooper et al., 2005, 2012) and stability (Hautier et al., 2015; Tilman & Downing, 1994) have been confirmed by a large number of studies (e.g., Hector & Bagchi, 2007; Poorter et al., 2017; Tilman et al., 2006, 2014; and reviewed by van der Plas, 2019). The positive BEF relationships have been confirmed in both theoretical (Turnbull et al., 2016) and empirical studies (Liang et al., 2016; Mensah et al., 2018; Venail et al., 2015) at small spatial scales (usually smaller than 0.1 ha), indicating that species diversity increases the opportunities for asynchronous and compensatory dynamics of species, thus improving ecosystem functioning (Isbell et al., 2011, 2015; Loreau, 2010). Nevertheless, why and how the relationship between biodiversity and ecosystem functioning changes with scale is one of the most controversial and contrasting topics (Gonzalez et al., 2020; Reu et al., 2022; Thompson et al., 2018, 2021).

Niche complementarity and selection hypothesis are two main, but not mutually exclusive, mechanisms to explain the positive relationships between biodiversity and ecosystem functions (e.g., productivity, carbon storage, and aboveground biomass; Grime, 1998; Loreau & Hector, 2001). The complementarity hypothesis states that diverse species assemblages have a greater variety of functional traits and can thus enhance resource use efficiency and nutrient retention (Morin et al., 2011), thereby increasing ecosystem functioning through interspecific facilitation, niche partitioning, or differentiation (Finke & Snyder, 2008; Hooper et al., 2005; Mensah et al., 2018; Tilman, 1999; Tilman et al., 2001), or through less harmful effect of herbivory/predation (Barry et al., 2019). The selection hypothesis states that diverse communities (i.e., higher diversity stands) increase the probability of including high‐functioning and/or ecologically important species (Cardinale et al., 2007; Loreau & Hector, 2001; Tilman et al., 2014). Thus, selection hypothesis are partially explained by the “mass‐ratio hypothesis” (Grime, 1998), which links the function of an ecosystem with the functional traits of the dominant species and predicts that ecosystem functions are driven by the traits of the most dominant species in the community (Mokany et al., 2008). Much debate on the mechanisms behind the observed positive BEF relationships has focused on whether diversity effects are driven by niche partitioning and facilitation (Tilman et al., 1997) or by the selection of one or more highly productive or high‐biomass species (Cardinale et al., 2012; Loreau & Hector, 2001). Some authors argued that the net effect of biodiversity on ecosystem functions is determined by both complementary and selection effects (Mokany et al., 2008; Tilman, 1996). The relative contribution of different mechanisms may vary with climatic conditions, forest types, and developmental stages (Ammer, 2019; Forrester & Bauhus, 2016). Thus, the mechanism of the BEF relationship needs to be further studied on bioclimatic gradients with different species composition and environmental conditions (e.g., different forest types). Moreover, it is especially not clear whether the relative importance of complementarity and selection effects vary with the spatial scale.

The spatial scale generally has a profound influence on ecological phenomena, as patterns apparent at one scale can collapse to noise when viewed at larger spatial extents (Hewitt et al., 2010). The relationship between biodiversity and ecosystem functions may theoretically vary with spatial scale (Gonzalez et al., 2020; Luo et al., 2019; Poorter et al., 2015). However, scaling the BEF relationships up to larger spatial scales in natural forests remains a challenge (Thompson et al., 2018), because most of the studies involved in the meta‐analysis have generally been limited to small spatial grains (i.e., the small size of the sampling unit or quadrat; typically, smaller than 0.1 ha) (Gamfeldt et al., 2013; Vilà et al., 2007). Based on observational data from temperate and tropical forests, Chisholm et al. (2013) found that species richness and biomass were positively related at small spatial scales (0.04 ha), whereas at larger spatial scales (1 ha), there was no consistent relationship between species richness and biomass, probably as a result of a saturation effect. Will the positive correlation between biodiversity and ecosystem functions persist at larger spatial scales (e.g., 0.25 and 1 ha) and in hyperdiverse communities (e.g., in the tropical rainforest), where numerous species could have functional redundancy?

Biodiversity exhibits inherent multiscale characteristics, represented by α diversity, β diversity, and γ diversity. However, in BEF studies, there has been insufficient focus on other aspects of biodiversity, such as β diversity, which pertains to spatial heterogeneity in species composition (Feizabadi et al., 2021). It is hypothesized that as spatial extent increases, even in situations with low α‐diversity and inability of species to coexist, β‐diversity can enhance ecosystem functioning through spatial niche complementarity during the sorting of species across environmental gradients (Reu et al., 2022; Thompson et al., 2021). New evidence from experimental (Pasari et al., 2013), theoretical (Pedro et al., 2016), and observational studies (Hautier et al., 2018) showed that β diversity can contribute toward simultaneously supporting single and/or multiple functions. Few authors even suggested that the BEF studies in the framework of β diversity have the potential to improve the prediction of natural and anthropogenic effects on diversity and ecosystem functions (Mori et al., 2018). Despite this perspective, the crucial role of β diversity in maintaining ecosystem functions has received much less attention compared with α diversity in BEF studies. Therefore, the evidence for the relationship between β diversity and ecosystem functions is lacking, and that is especially true for observational field data in naturally assembled forest communities. Furthermore, Baselga (2010) proposed that the total amount of β diversity can be decomposed into two separate components, spatial species turnover and nestedness of assemblages, which result from two antithetic processes, namely species replacement and species loss, respectively. Few previous studies suggested that the niche complementarity effect may link to the turnover component of β diversity (Mokany et al., 2015). While the nestedness components highlight the importance of dominant species (Hautier et al., 2018) at larger scales, which could be explained by the selection effects (Omidipour et al., 2021). Although this viewpoint is theoretically sound, it has not been substantiated by sufficient empirical and observational studies in different forest types, especially in temperate and tropical forests, where species richness varies greatly.

We here mainly focus on the relationships between biodiversity (α diversity and β diversity) and ecosystem function (the aboveground biomass) in temperate and tropical forests, by addressing the following specific questions: (1) Is there a positive relationship between diversity (species richness, functional diversity, or functional dominance) and aboveground biomass? (2) How do these relationships vary with spatial scales and forest types? That is, whether the relative importance of complementarity and selection mechanisms varied with different spatial scales and forest types. (3) Is there a relationship between β diversity and aboveground biomass? If so, is β diversity a better predictor of ecosystem function than α diversity (e.g., species richness)? What are the relationships between aboveground biomass and turnover and nestedness components of β diversity?

## 2 METHODS

### 2.1 Study areas and FDPs

This study was carried out in two bioclimatic regions, including tropical and temperate forests in China. In each region, two 6‐ha (300 m × 200 m) forest dynamics plots (FDPs) were selected, which we refer to Jianfengling and Bawangling FDPs (abbreviated as JFL and BWL, respectively) in the tropical forest, and Jiaohe and Lushuihe FDPs (abbreviated as JH and LSH, respectively) in the temperate forest. The JFL FDP, which represents the old‐growth tropical montane rainforest, is located in the Jianfengling National Nature Reserve in the south‐western region of Hainan Island, China (Xu et al., 2015; Zang et al., 2019). The BWL FDP, which represents the old‐growth tropical montane rainforest, is located in the Bawangling National Nature Reserve in the south‐western region of Hainan Island, China (Ding et al., 2012). The JH FDP is located in a forest area under the jurisdiction of the Jiaohe Administrative Bureau in Jiaohe, Jilin Province, in northeastern China. The forest type is a typical old‐growth temperate mixed broadleaf–conifer forest (Yao et al., 2020). The LSH FDP is located in a typical old‐growth temperate mixed broadleaf–conifer forest in Dongsheng Forest Farm, Baishan, Jilin Province, in northeastern China. The main features of FDPs are summarized in Table 1.

**TABLE 1:** Locations, climatic conditions, and overall statistics of the forest dynamics plots (FDP).

Forest types	Forest dynamics plots (FDPs, 6 ha)	Coordinates (deg.)	Climate	Temp. (°C)	Rainfall (mm)	No. of species	No. of individuals	Plot size (m × m)
Tropical montane rainforest	Jianfengling (JFL)	18.63 N 108.94 E	Tropical	24.5	2265.8	210	11,096	300 × 200
	Bawangling (BWL)	19.07 N 109.67 E	Tropical	23.6	1751	270	8902	300 × 200
Temperate mixed broadleaf–conifer forest	Jiaohe (JH)	43.75 N 127.33 E	Temperate	3.8	696	26	4057	300 × 200
	Lushuihe (LSH)	41.50 N 127.76 E	Temperate	2.9	894	30	3882	300 × 200

Abbreviations: Rainfall, mean annual rainfall (mm); Temp., mean annual temperature (°C).

### 2.2 Experimental design and data collection

All FDPs were established according to the standard of the Forest Global Earth Observatory (ForestGEO, https://forestgeo.si.edu/). The census methodology was identical for all FDPs: All woody stems with a diameter at breast height (dbh) of 1 cm or larger were spatially mapped, measured, and identified to the species level, and tagged. Each FDP was subdivided into nonoverlapping quadrats at three spatial grains: 20 m × 20 m (0.04 ha), 50 m × 50 m (0.25 ha), and 100 m × 100 m (1 ha), for analyzing the scale‐dependent relationships between diversity and ecosystem function. Only individuals with dbh ≥5 cm were used in calculations of the aboveground biomass (AGB) in this study. For each species, we obtained values of five plant functional traits: specific leaf area (SLA, cm^2^ g^−1^), leaf dry‐matter content (LDMC, g g^−1^), leaf nitrogen content (LNC, g kg^−1^), leaf phosphorus content (LPC, g kg^−1^), and wood density (WD, g cm^−3^). Plant leaf traits are related to a species' resource use efficiency (Wright et al., 2004), and the functional traits in stem tissues (e.g., wood density) are recognized as powerful indicators of plant mechanical strength and directly affect AGB (Messier et al., 2017). Details on the functional traits sampling procedures and measurements are given by Bu et al. (2019), Yu et al. (2020) and Ding and Zang (2021).

We used the same equation to calculate AGB in different climatic zones to ensure comparability. According to Chave et al. (2005), we first classified the FDPs as “dry” (<1500 mm year^−1^ precipitation) or “moist” (1500–3500 mm year^−1^). We then calculated AGB for individual, live trees using allometric equations for dry and moist forests from Chave et al. (2005). Total AGB for each quadrat at each spatial grain was calculated by summing AGB for all stems in the quadrat.

### 2.3 Statistical analysis

For each FDP, we measured three dimensions of tree diversity at each quadrat: species diversity, functional diversity, and functional dominance. Species diversity was characterized by species richness (*S*) and Pielou's evenness (*J*) for each quadrat at each spatial grain. Functional diversity describes the variability in functions or characteristics of the species in a community. As functional diversity metrics, we calculated the functional richness (FRic), functional evenness (FEve), functional divergence (FDiv) (Mason et al., 2005), and functional dispersion (FDis) (Laliberté & Legendre, 2010) for each quadrat at each spatial grain. As functional dominance measures the degree to which a trait is more numerous (or dominant) than others, which is closely linked to the selection effect, we thus estimated the community weight mean (CWM) for the five plant functional traits (SLA, LDMC, LNC, LPC, and WD) to characterize the functional dominance. The species diversity was calculated using the vegan package (Oksanen et al., 2022), and functional diversity and functional dominance were calculated using the FD package (Laliberté et al., 2014; Laliberté & Legendre, 2010) in the R statistical software package (R Core Team, 2021).

Unlike α diversity, β diversity refers to the difference in species composition between two sites or communities, we thus extracted 6100 m × 100 m subsamples from the 6 ha FDPs. We then calculated β diversity for each subsample at the 20 m × 20 m spatial extent, according to the Baselga (2010). The β diversity (β_JAC_) and its components: turnover (β_JTU_) and nestedness (β_JNE_) were calculated using multiple‐site dissimilarity by “betapart” package (Baselga et al., 2021) as follows:

$$\beta_{\mathrm{JAC}}=\frac{\left[\sum_{i<j}\min\left(b_{\mathrm{ij}}∙b_{\mathrm{ji}}\right)\right]+\left[\sum_{i<j}\max\left(b_{\mathrm{ij}}∙b_{\mathrm{ji}}\right)\right]}{\left[\sum_{i}S_{i}-S_{T}\right]+\left[\sum_{i<j}\min\left(b_{\mathrm{ij}}∙b_{\mathrm{ji}}\right)\right]+\left[\sum_{i<j}\max\left(b_{\mathrm{ij}}∙b_{\mathrm{ji}}\right)\right]}$$

$$\beta_{\mathrm{JTU}}=\frac{2\left[\sum_{i<j}\min\left(b_{\mathrm{ij}}∙b_{\mathrm{ji}}\right)\right]}{\left[\sum_{i}S_{i}-S_{T}\right]+2\left[\sum_{i<j}\min\left(b_{\mathrm{ij}}∙b_{\mathrm{ji}}\right)\right]}$$

$$\beta_{\mathrm{JNE}}=\frac{\left[\sum_{i<j}\max\left(b_{\mathrm{ij}}∙b_{\mathrm{ji}}\right)\right]-\left[\sum_{i<j}\min\left(b_{\mathrm{ij}}∙b_{\mathrm{ji}}\right)\right]}{\left[\sum_{i}S_{i}-S_{T}\right]+\left[\sum_{i<j}\min\left(b_{\mathrm{ij}}∙b_{\mathrm{ji}}\right)\right]+\left[\sum_{i<j}\max\left(b_{\mathrm{ij}}∙b_{\mathrm{ji}}\right)\right]}$$

where *S*_*i*_ and *S*_*T*_ are the number of species in site *i* and *T*, *b*_*ij*_ and *b*_*ji*_ are the number of species that are only found in sites *i* and *j*, respectively. β_JAC_ is the total β diversity calculated from the Jaccard coefficient of dissimilarity, β_JTU_ and β_JNE_ are turnover and nestedness components of β diversity, respectively.

We used linear mixed model analyses to examine the combined effects of species diversity, functional diversity, and functional dominance on aboveground biomass (Table S1). We acknowledged that the quadrats within a given FDP may be expected to be similar and should not be considered independent (Figure S1). To accounted for the spatial autocorrelation, the polynomial trend surface techniques were used for spatial detrending (Borcard et al., 2018). We built a third‐degree polynomial function of the *X* and *Y* coordinates of the quadrats to account for the spatial autocorrelation of all variables. Then, the residuals were retained as the detrended dataset for the subsequent analysis (Borcard et al., 2018; Hao et al., 2020; Tan et al., 2019). All explanatory variables were standardized to a mean of 0 and SD of 1 before conducting the regression analysis. Model selection was conducted, comparing all possible models and including all variables, with the “MuMIn” package (Bartoń, 2020) in R. Standardized regression weights (coefficients) were applied to compare the relative importance of different variables on AGB, with the “apaTables” package (Stanley, 2021). The relationships between β diversity as well as their components (i.e., turnover and nestedness) and aboveground biomass were analyzed using the same statistical analysis.

## 3 RESULTS

**FIGURE 1:**
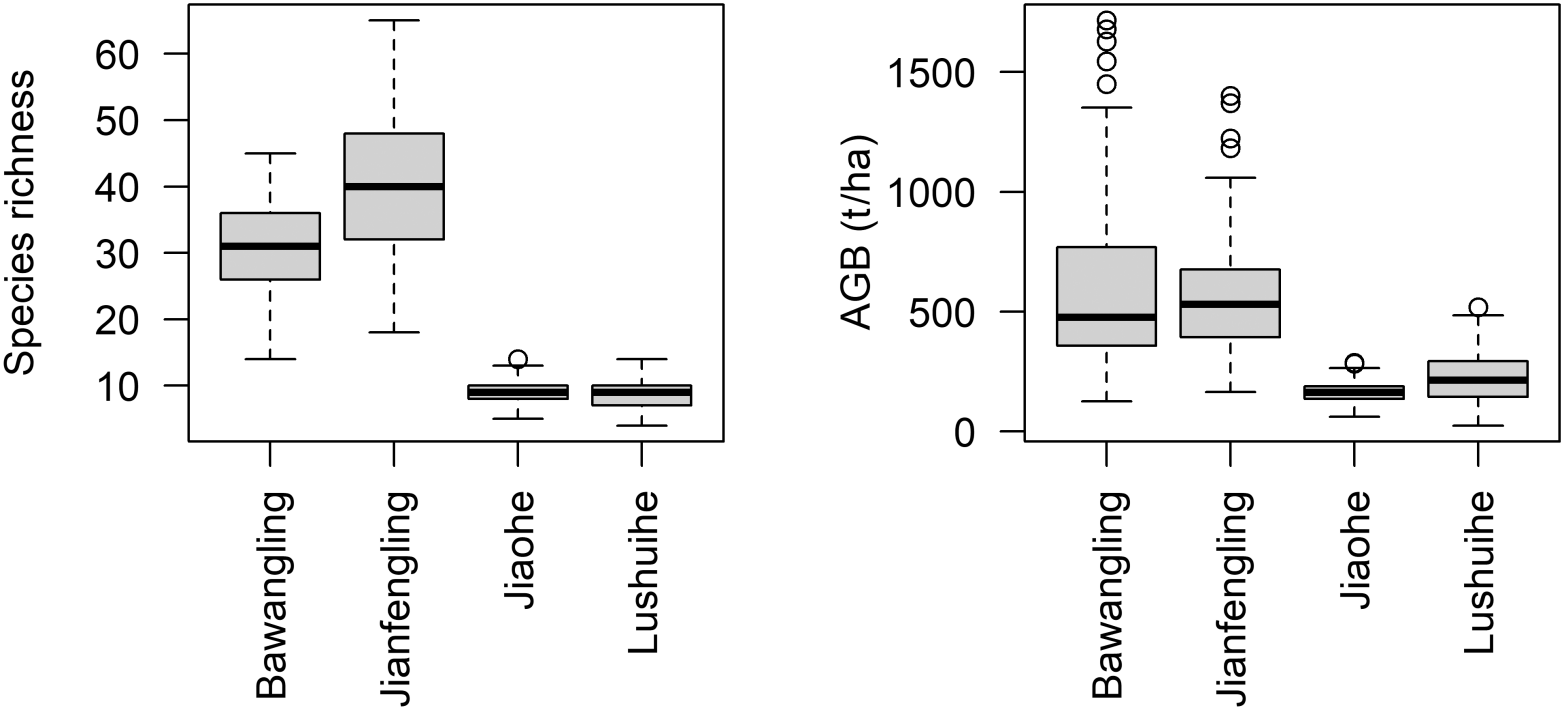
Distribution of plot (a) species richness (at 0.04 ha level) and (b) aboveground biomass (AGB, t ha^−1^) at each FDP.

The number of tree species per quadrat (0.04 ha) ranged from 4 to 65; averagely, 30.9, 40.0, 9.0, and 8.7 in BWL, JFL, JH, and LSH FDP, respectively (Figure 1a). On average, the aboveground biomass was 592.2, 567.9, 160.4, and 223.4 t ha^−1^ in BWL, JFL, JH, and LSH FDP, respectively (Figure 1b). Species richness (two‐sample *t*‐test: *t* = 47.32, df = 322.49, *p* < .001) and aboveground biomass (*t* = 22.55, df = 349.98, *p* < .001) in the tropical forest FDPs were significantly higher than those in the temperate forest (Figure 1).

**FIGURE 2:**
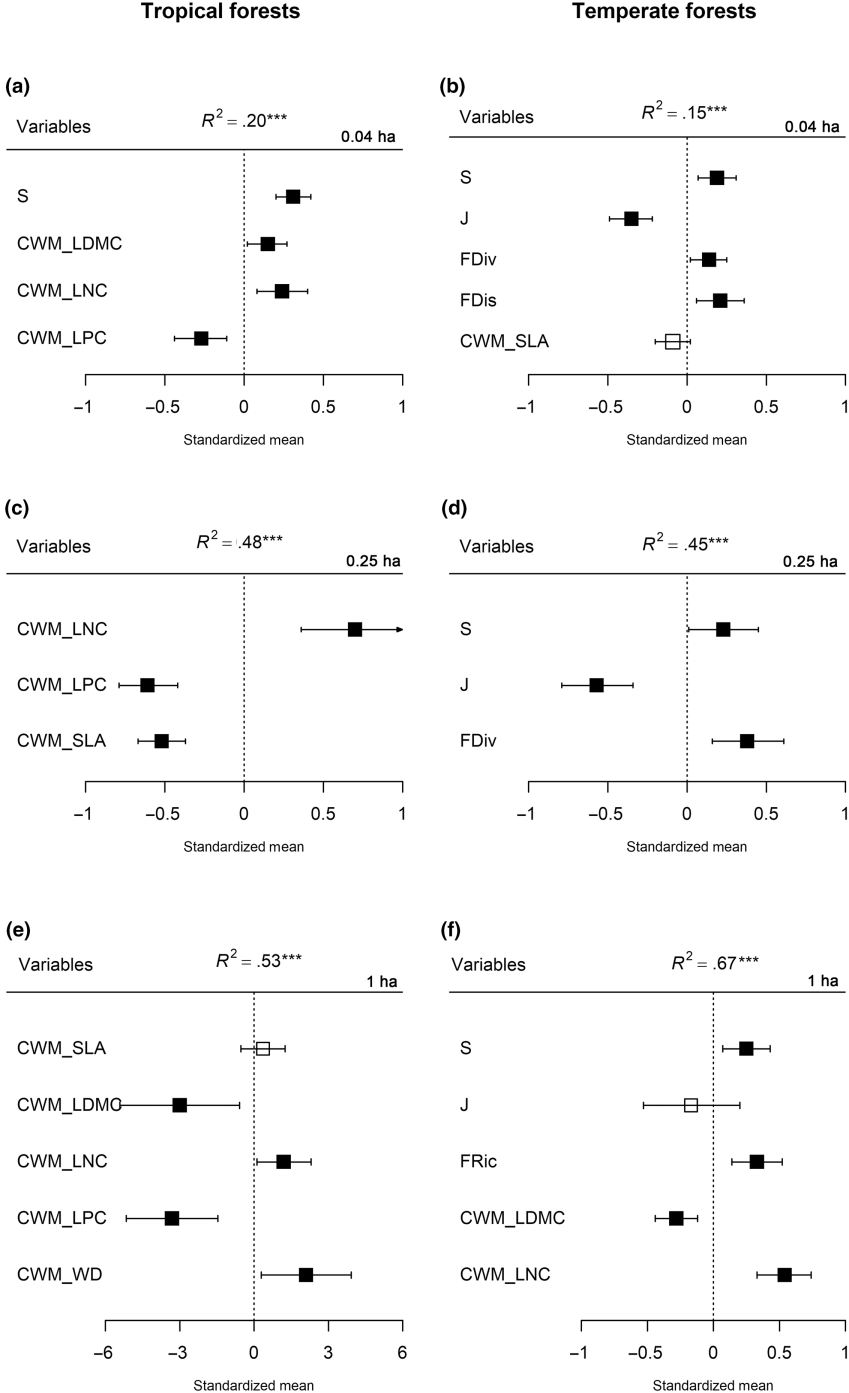
Results of linear mixed‐effect model for the effect of species diversity, functional diversity and functional dominance on aboveground biomass across tropical and temperate forests at the spatial grains of 0.04, 0.25 and 1 ha. FDP was included as a random effect in the models. CWM, community weight means; FDis, functional dispersion; FDiv, functional divergence; FEve, functional evenness; FRic, functional richness; J, Pielou's evenness; LDMC, leaf dry‐matter content; LNC, leaf nitrogen content; LPC, leaf phosphorus content; S, species richness; SLA, specific leaf area; WD, wood density. Only the variables retained after the model selection are shown. Black square—significant effect on AGB, white square—nonsignificant effect on AGB. ***p < .001.

The relationships between aboveground biomass and species diversity, functional diversity, and functional dominance (represented by CWM) varied with forest types and spatial grains (Figure 2, Table S1). In the tropical forest, the effect of species richness on aboveground biomass decreased with the spatial grains, while the functional dominance played dominant roles in explaining variation in AGB at larger spatial grains (e.g., 0.25 and 1 ha; Figure 2a,c,e). In the temperate forest, the relationship between diversity (taxonomic and functional diversity) and aboveground biomass showed a significant positive correlation at all spatial grains (Figure 2b,d,f). The functional diversity (FDiv, FRic) were positively correlated with aboveground biomass and had more explanatory ability in determining AGB than functional dominance at 0.04 and 0.25 ha grains (Figure 2b,d).

Our results indicated that at a very small spatial grain (0.04 ha), species richness was significantly and positively correlated with aboveground biomass in both tropical and temperate forests (Figure 2a,b). At a larger spatial grain (0.25 and 1 ha), there was no correlation between species diversity and aboveground biomass in the tropical forest (Figure 2e), while there was still a significant positive correlation between species richness and aboveground biomass in the temperate forest (Figure 2f). Overall, the positive effects of species diversity on aboveground biomass were stronger in temperate forests and weaker in tropical forests. However, the effect of functional dominance (CWM) was stronger in tropical than in temperate forests.

**FIGURE 3:**
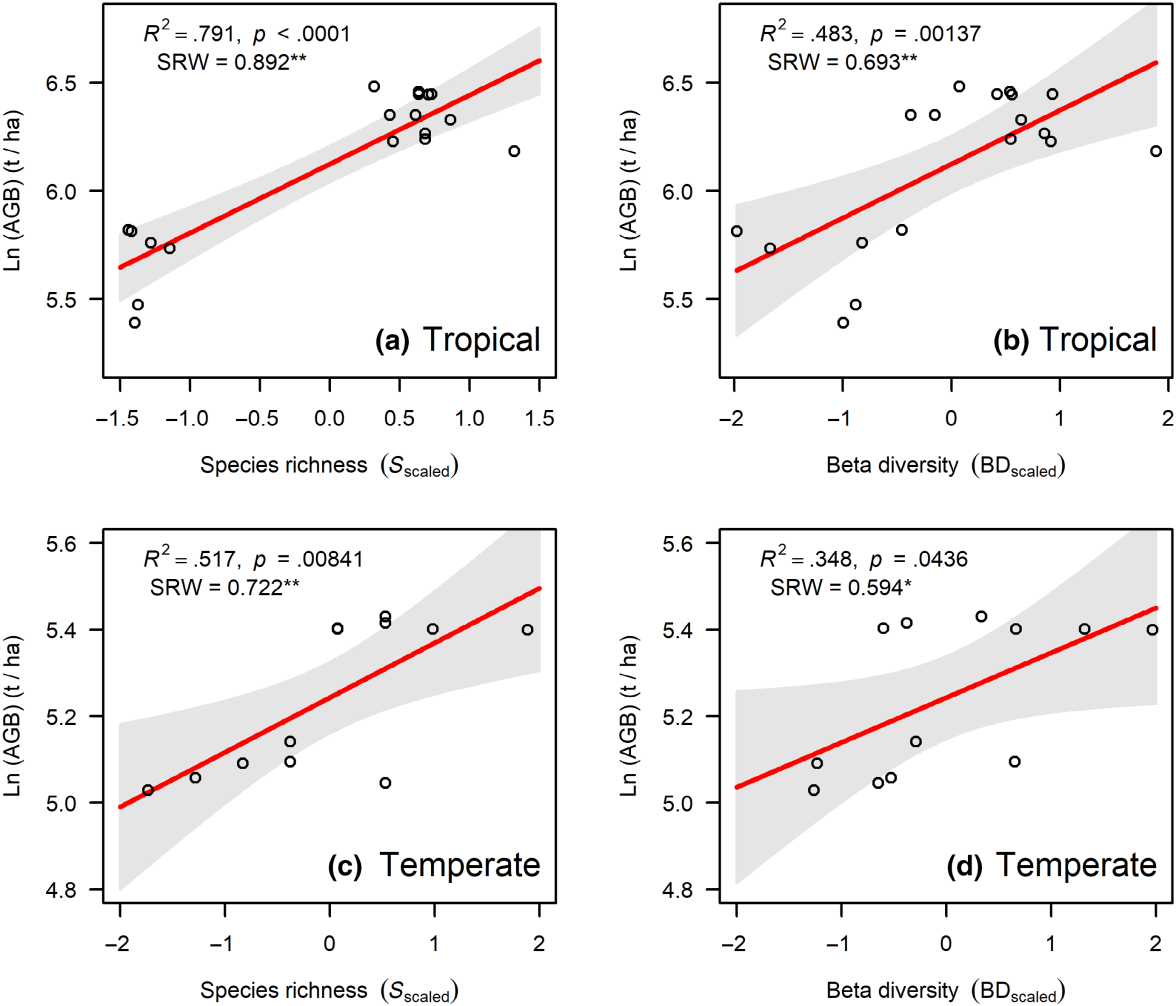
Results of the relationship between species richness (a, c) and β diversity (b, d) with aboveground biomass in tropical and temperate forests. **p < .01; *p < .05. SRW, indicates the standardized regression weights. Shaded area gives the 95% confidence intervals. The β diversity were calculated at the 20 m × 20 m spatial extent (more details see the Section 2.3).

**FIGURE 4:**
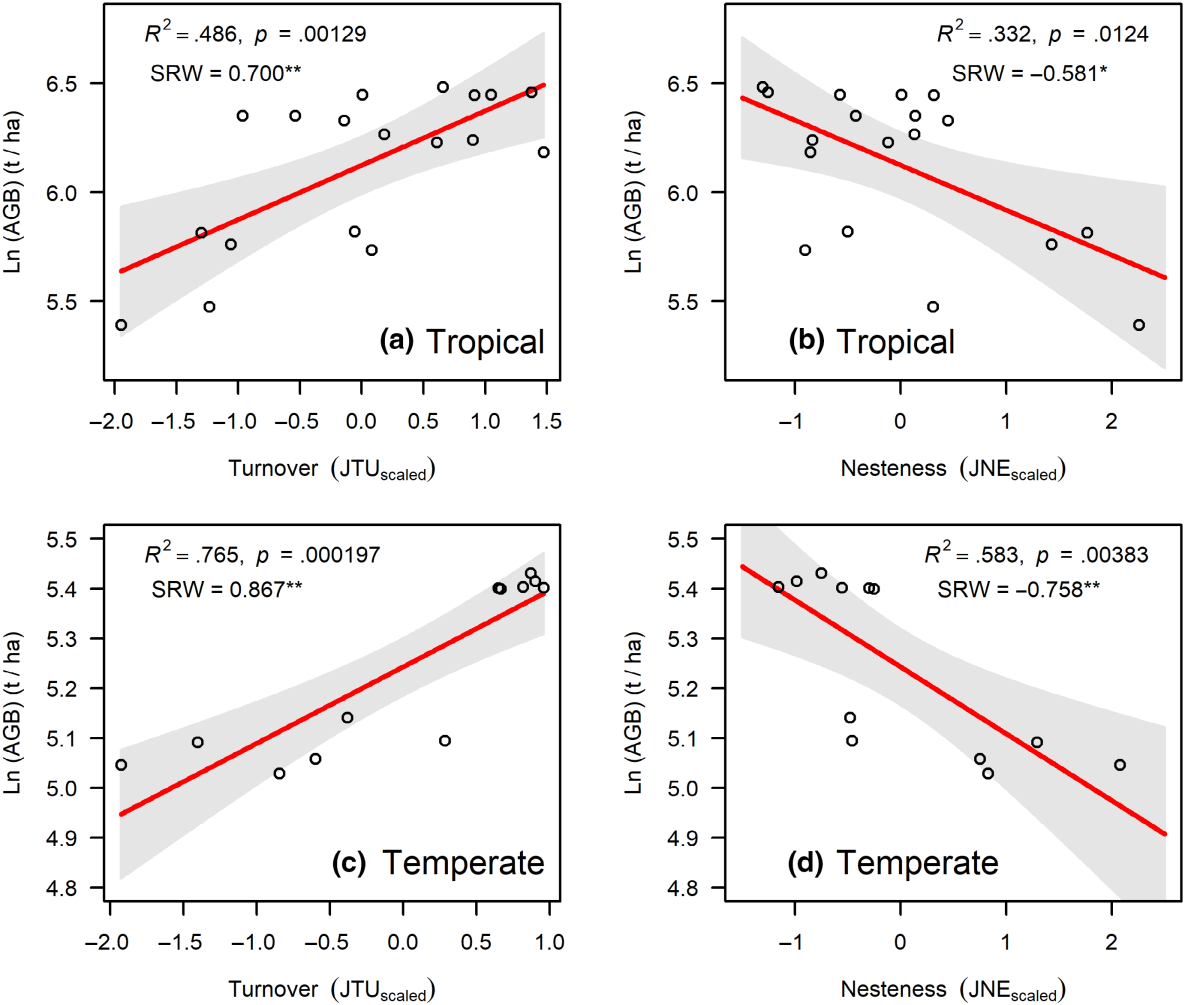
Results of the relationship between turnover (a, c) and nestedness components (b, d) of β diversity with aboveground biomass in tropical and temperate forests. **p < .01; *p < .05. SRW, indicates the standardized regression weights. Shaded area gives the 95% confidence intervals.

β diversity was significantly and positively correlated with aboveground biomass in both tropical and temperate forests (Figure 3b,d), suggesting that as α diversity, β diversity was also a good predictor of aboveground biomass (Tropical: *R* ^2^ = .483; Temperate: *R* ^2^ = .348). However, the explanatory power of the model (*R*‐squared) and the standardized regression weight (SRW) coefficients indicated that α diversity is more efficient than β diversity in explaining the variation of aboveground biomass in our FDPs (Figure 3). Moreover, the species turnover component of β diversity was positively correlated with aboveground biomass (Figure 4a,c), while the nestedness component was negatively correlated with aboveground biomass (Figure 4b,d).

## 4 DISCUSSION

### 4.1 The scale dependency of BEF relationships

Understanding how biodiversity maintains and promotes ecosystem function has received considerable attention and has been a subject of controversy for more than three decades (Adler et al., 2011; Grime, 1973; Lasky et al., 2014). Hundreds of biodiversity experiments have accumulated evidence that species richness enhances ecosystem functioning (e.g., productivity, biomass, carbon sequestration, habitat provision, water and soil protection) (Albrecht et al., 2021). Key questions remain as to whether findings from experimental communities and small‐scale studies shed light on BEF relationships in naturally assembled communities and at large spatial scales (Reu et al., 2022). Our empirical study found a positive correlation between species richness and aboveground biomass, especially at a small spatial scale. In temperate forests with relatively low species richness, species diversity plays a consistently important role in aboveground biomass (Figure 2b,d,f). In the tropical forest, however, the relationship between species richness and aboveground biomass showed neutral correlation at a relative larger spatial scale. We hence hypothesized that the niche complementarity effect was particularly significant at small spatial scales due to small sample area and low species richness. In other words, there was a significant positive relationship between diversity and aboveground biomass at small scales, possibly due to the addition of any one species might have a large impact on productivity or biomass. At relative larger spatial scales, however, the relationship between diversity and aboveground biomass is insignificant or nonexistent, which may be caused by saturation effect or functionally redundant at a large spatial scale (Cardinale et al., 2012; Liang et al., 2015).

### 4.2 The BEF relationships varied with different forest types

The correlation between species richness and aboveground biomass may also be due to the influence of environmental variables. In temperate forests, there is a consistent significant positive correlation between species diversity and aboveground biomass at any spatial scale (Figure 2b,d,f). Species diversity indeed had a significant effect on biomass and productivity in the temperate mixed broadleaf–conifer forest of northeastern China, even when the effects of covarying environmental factors were included (Wu, 2018). However, in tropical forests with high habitat heterogeneity, environmental factors should be considered in the BEF studies (Ding & Zang, 2021). We thus acknowledge that one of the deficiencies of our study is the unavailability of environmental data, which results in failing to account for the potential influence of environmental heterogeneity on the relationship between species diversity and aboveground biomass. Future studies should simultaneously focus on how do abiotic and biotic variables directly or indirectly affect ecosystem functions, in particular, how do their relative roles vary with the spatial scales.

Our results indicated that the relationships between aboveground biomass and species diversity, functional diversity, and functional dominance varied with forest types (i.e., tropical vs. temperate forests). For instance, the community‐weighted trait means (CWMs) explained more variation in AGB than did species diversity and functional diversity at 0.25 and 1 ha grains in tropical forests. This suggested that the selection effect may play a dominant role in aboveground biomass in tropical forests. Moreover, the larger the spatial scale, the more obvious the leading roles of the selection effect on the aboveground biomass in tropical forests. In temperate forests, however, species richness showed positive correlation with aboveground biomass at any spatial grains, suggesting that the role of species diversity in temperate forests was always important to aboveground biomass. These results indicated that niche complementarity effects might be the dominant mechanism in determining the aboveground biomass in temperate forests. Some studies suggested that niche complementarity is less important in stable and high‐yield environments where interactions between species are dominated by competition rather than complementarity, while the positive effects of niche complementarity on productivity, biomass, or C storage are particularly important in unstable and stressful environments (Paquette & Messier, 2011). Species richness and functional diversity may thus play a larger role in driving AGB in harsher environments (e.g., boreal forests). In hyperdiverse tropical forests, however, diversity might be less relevant to AGB partly due to a saturating effect of diversity (Ruiz‐Jaen & Potvin, 2011). It should be noted that although the effect of species diversity on AGB was strong and positive in our temperate forest, it cannot be ruled out that the greater diversity effect in the temperate forest is simply the result of relatively lower species richness.

### 4.3 The contributions of species spatial heterogeneity to aboveground biomass

We found a significant positive correlation between β diversity and aboveground biomass (Figure 3b,d). However, α diversity predicts the variation in aboveground biomass more effectively than β diversity (Figure 3), which contrasted with a few previous studies (Omidipour et al., 2021; Pedro et al., 2016). Our study indicated that the species turnover and nestedness component of β diversity were positively and negatively correlated with aboveground biomass, respectively (Figure 4). Contrasting to our findings, Omidipour et al. (2021) found that the AGB was significantly and positively related to the both turnover and nestedness components of β diversity in Mediterranean rangeland. The most likely explanation is that the species turnover should be more likely to occur compared to nestedness at a relatively small local scale (i.e., plot scale; Baselga, 2010, 2012). We thus inferred that the positive effects of β diversity on aboveground biomass in our forests were mainly due to the spatial species turnover at the plot scale. One possible explanation is that different species demonstrate distinct performances under varying environmental conditions. They have the ability to complement each other in utilizing available resources, thereby contributing to an enhanced occupation of niche space across different locations (Mori et al., 2018). Therefore, the complementarity effect of diversity for ecosystem functions could be linked to the turnover component of β‐diversity.

Although the results of our study supported a positive correlation between β diversity and aboveground biomass, there are several things worth noting. First of all, although the FDP area used in this study is relatively larger than the plot size that was usually studied before (e.g., 0.04 ha), the spatial scale considered in present study is still on the local scale (that is, at the plot scale). Second, we investigated the relationship between β diversity and the individual ecosystem function (i.e., aboveground biomass), rather than the relationship between β diversity and multifunctionality at the landscape level as few previous studies have shown (Mori et al., 2016; van der Plas et al., 2016). Spatial heterogeneity of community species composition may contribute to ecosystem functions through two main mechanisms (Hautier et al., 2018). First, if different species have various functions in different locations, then the dissimilarity in functionally important species can maintain ecosystem function in different landscapes. In other words, higher β diversity is positive for the maintenance of ecosystem multifunctionality. For instance, α diversity had strong positive effects on most individual functions and multifunctionality, and the positive effects of β diversity will be more pronounced when multiple functions are considered simultaneously, compared to when only a single function is taken into account (Pasari et al., 2013). We thus inferred that it is possible to underestimate the positive effect of β diversity on ecosystem function when only aboveground biomass is considered. This may explain why β diversity did not explain variation in AGB better than α diversity in our study. Future studies should focus on the relationships between β diversity and multiple ecosystem functions simultaneously (multifunctionality), and we assume that the role of β diversity in maintaining multifunctionality should increase. The second plausible mechanism for β diversity to promote ecosystem functions is that dissimilarity in species composition among local communities can influence ecological interactions. For instance, local communities that provide habitats for insects may provide pollination and pest control for neighboring communities, thereby facilitating ecosystem functions at local and landscape scales (Tscharntke et al., 2005). The “insurance effects of β diversity” may be significant only in the spatiotemporal interactions between communities that are not randomly distributed in large spatial regions (e.g., bioclimatic regions, macroecological scales) (Mokany et al., 2015). Observational data from real and simulated artificial landscapes suggested that the relationships between β diversity and multifunctionality were always positive only at the landscape level (Mori et al., 2016; van der Plas et al., 2016). However, the local level in our study may be another plausible reason why the ability of β diversity to explain the variation in AGB was underestimated. Third, the enhanced association between β diversity and biomass at larger spatial scales may be attributed to the escalated levels of environmental heterogeneity (e.g., topographic and resource) at those scales (Reu et al., 2022; van der Plas et al., 2023). Environmental heterogeneity leads to increased variation in species composition by favoring different species that are best adapted to local environmental conditions (known as species sorting; Leibold et al., 2004). This process is anticipated to enhance ecosystem functions such as productivity because species are filtered into environments where their traits can most efficiently convert resources into biomass (Hammill et al., 2018). Species composition turnover due to environmental heterogeneity can be viewed as a form of complementarity, where various species contribute to ecosystem functioning under different conditions (Vasseur & Yodzis, 2004).

### 4.4 Potential for future studies

Recent quantitative studies pointed out that the observed patterns of the relationship between β diversity and ecosystem function were not always consistent (reviewed by Mori et al., 2018; van der Plas et al., 2023). This may be partly due to the different definitions and measures of β diversity in different studies, the possible dependence between α diversity and β diversity, and/or lacking the gradient of spatial scales (e.g., sample units area). Therefore, it is not possible to derive a generalized theory of the role of β diversity in ecosystem functions. Future studies on β diversity and ecosystem (multi‐) function should consider the following attractive aspects: First, studying the underlying ecological mechanisms of the spatial and temporal variations in local diversity (β diversity) can help to understand (and therefore manage) the relationship between biodiversity and ecosystem functions. That is, it is really fascinating to illuminate to what extent the key processes of driving β diversity are linked with the mechanisms underpinning biodiversity–ecosystem functioning relationships, and how the relative importance of these processes changes in space and time. Second, it is indeed important to consider the effects of biodiversity on ecosystem functions at multiple spatial scales. In general, the roles of β diversity and spatial scales in regulating the effects of biodiversity changes on ecosystem function is related to the changes in biodiversity at the local scale, where may lead to local changes in ecosystem function. Such changes can be extended to large‐scale changes in multiple ecosystem functions (Hautier et al., 2018; Mori et al., 2016). Third, trying to understand scale‐dependent effects of beta diversity on ecosystem functioning by looking at the different causes of changes in beta diversity. van der Plas et al. (2023) explored three scenarios that cause gradients in β‐diversity, namely (i) variation in abiotic heterogeneity, (ii) variation in habitat isolation that alters β‐diversity through changes in dispersal rates, and (iii) variation in species poor richness and thereby β‐diversity. Overall, to understand the importance of β‐diversity for the ecosystem functioning, we have to clarify why universally positive relationships should not be anticipated.

We investigated the relationships between β diversity and aboveground biomass just at the spatial grains of 0.04 ha, considering that the 6‐ha plot is not a large enough area to study the relationship at multiple spatial scales. We thus failed to clarify how do the relationships between β diversity as well as their components (i.e., turnover and nestedness) and aboveground biomass vary with spatial scales. In fact, there are two aspects of spatial scale: grain and extent. It is worth to explore the relationship between β diversity and ecosystem (multi‐)functionality: (1) at multiple spatial scales (i.e., spatial grain), and (2) at the scale of the regional or continental level (i.e., spatial extent). The Forest Global Earth Observatory (ForestGEO), previously known as Center for Tropical Forest Science (CTFS), is a global network of forest research sites and scientists dedicated to the study of tropical and temperate forest function and diversity (https://forestgeo.si.edu/). The multi‐institutional network supported long‐term data collection at 73 forest research sites across the Americas, Africa, Asia, Europe, and Oceania. The unprecedented network may support the potential scientists to explore the relationship between β diversity and aboveground biomass not only at multiple spatial scales, but also the relationship between β diversity and multifunctionality at the scale of the regional even to the continental level. Focusing on this not currently well‐examined dimension of biodiversity (i.e., β diversity) has the profound potential not only to help quantify the importance of biodiversity in maintaining (multiple) functioning but also to mechanistically uncover observed patterns of spatiotemporal variation in species assemblage and ecosystem function.

## 5 CONCLUSIONS

An unresolved concern in the field of the relationship between biodiversity and ecosystem function is whether BEF relationships as observed at small spatial scales, and their underlying mechanisms, are also present at the much larger spatial scales. Our results indicate that the BEF relationships, and their underlying mechanisms are scale‐dependent, meanwhile varied with different forest types. The roles of species spatial heterogeneity (i.e., β diversity) in maintaining the ecosystem functioning are the major research gap until now. Our study supported a positive correlation between β diversity and aboveground biomass, albeit at the local spatial scale. Moreover, we found that β diversity was weaker than α diversity in explaining variation in AGB in our forests. However, there is an emerging question, saying, whether α‐ or β‐diversity is the most important driver for ecosystem functioning, and which mechanisms drive BEF relationships at larger spatial scales. Anyway, β diversity should not be neglected in BEF explorations in the context of multifunctionality across large areas of spaces. Our study provides implications that it is important to conserve the biotic heterogeneity especially under the context of increasingly biotic homogenization.

## AUTHOR CONTRIBUTIONS

**Jie Yao:** Data curation (lead); formal analysis (lead); investigation (equal); methodology (equal); writing – original draft (lead). **Jihong Huang:** Data curation (equal); funding acquisition (lead). **Runguo Zang:** Conceptualization (lead); funding acquisition (equal); resources (lead); validation (lead); writing – original draft (equal).

## CONFLICT OF INTEREST STATEMENT

The authors declare no conflict of interest.

## Supporting information


**Data S1.** Supporting Information.

## Data Availability

Data available from the Dryad Digital Repository (https://doi.org/10.5061/dryad.z612jm6fs). The data that support the findings of this study are openly available in Dryad at https://doi.org/10.5061/dryad.z612jm6fs. https://datadryad.org/stash/share/QzMe9accpLS1DDmr1yaUn6ACqDO8fU‐20hpVbgAj5vY.
