# Supplementary material for: Alpha and beta diversity jointly drive the aboveground biomass in temperate and tropical forests
Source: Ecol Evol. 2023 Aug 31;13(9):e10487. doi: 10.1002/ece3.10487 (PMC10468913; doi:10.1002/ece3.10487)

Supporting Information

Species diversity and spatial heterogeneity jointly drive the aboveground biomass in temperate and tropical forests

Jie Yao[1,2]; Jihong Huang[1,2]; Runguo Zang[1,2]

1 Ecology and Nature Conservation Institute, Chinese Academy of Forestry, Key Laboratory of Forest Ecology and Environment of National Forestry and Grassland Administration, Beijing 100091, China

2 Co-Innovation Center for Sustainable Forestry in Southern China, Nanjing Forestry University, Nanjing 210037, China

Corresponding author: Runguo Zang,

No. 2 Dongxiaofu, Haidian District, Beijing 100091, China

E-mail address: zangrung@caf.ac.cn.

Table S1 Results of linear mixed-effect model for the effect of species diversity, functional diversity and functional dominance on aboveground biomass across tropical and temperate forests at the spatial grains of 0.04 ha, 0.25 ha and 1 ha. FDP was included as a random effect in the models. S: species richness, J: Pielou's evenness; FRic: functional richness, FEve: functional evenness, FDiv: functional divergence, FDis: functional dispersion; CWM: community weight means; SLA: specific leaf area, LDMC: leaf dry-matter content, LNC: leaf nitrogen content, LPC: leaf phosphorus content, WD: wood density. Only the variables retained after the model selection are shown. * p < 0.05, ** p < 0.01, *** p < 0.001.

**Spatial grains**	**Tropical forest**					**Temperate forest**				
	**Variables**	**Estimate**	**Std. Error**	***z*-value**	**Pr(>|z|)**	**Variables**	**Estimate**	**Std. Error**	***z*-value**	**Pr(>|z|)**
**0.04 ha**	Intercept	3.02	0.03	121.02	< 0.001***	Intercept	1.95	0.02	89.85	< 0.001***
	S	0.32	0.06	5.51	< 0.001***	S	0.21	0.06	3.22	0.001 **
	CWM_LNC	0.24	0.08	2.94	0.003 **	J	-0.35	0.07	-5.20	< 0.001***
	CWM_LPC	-0.28	0.09	-3.24	0.001 **	FDis	0.22	0.08	2.80	0.005 **
	CWM_LDMC	0.15	0.07	2.34	0.02 *	FDiv	0.14	0.06	2.40	0.017 *
						CWM_SLA	-0.09	0.06	-1.54	0.114
**0.25 ha**	Intercept	4.95	0.03	197.02	< 0.001***	Intercept	3.84	0.02	190.12	< 0.001***
	CWM_SLA	-0.62	0.21	-2.99	0.005 **	S	0.23	0.11	2.10	0.04 *
	CWM_LNC	0.76	0.18	4.12	< 0.001***	J	-0.64	0.13	-5.06	< 0.001***
	CWM_LPC	-0.68	0.21	-3.16	0.002 **	FDiv	0.38	0.11	3.39	0.001 **
**1.00 ha**	(Intercept)	6.35	0.02	362.43	< 0.001***	(Intercept)	5.24	0.01	792.62	< 0.001***
	CWM_SLA	0.04	0.06	0.78	0.473	S	0.07	0.01	4.48	0.003 **
	CWM_LDMC	-0.31	0.10	-3.20	0.024 *	J	-0.03	0.02	-2.65	0.143

	CWM_LNC	0.26	0.13	2.08	0.042*	FRic	0.11	0.02	5.20	0.001**
	CWM_LPC	-0.52	0.15	-3.51	0.017 *	CWM_LDMC	-0.12	0.02	-5.23	0.001**
	CWM_WD	0.15	0.05	2.78	0.038 *	CWM_LNC	0.23	0.04	6.13	< 0.001***

Fig. S1 The spatial correlograms of univariate quantitative variables at the spatial of 20 × 20 m quadrat in FDPs. Moran's I statistics were used to measure spatial correlation of the explanatory and response variables. No.sp, number of species per quadrat; FRic, functional richness; FEve, functional evenness; FDiv, functional divergence; FDis, functional dispersion; the community weight mean (CWM) for the five plant functional traits (SLA, LDMC, LNC, LPC, and WD); agb, aboveground biomass per quadrat.

(a) Bawangling forest dynamics plot

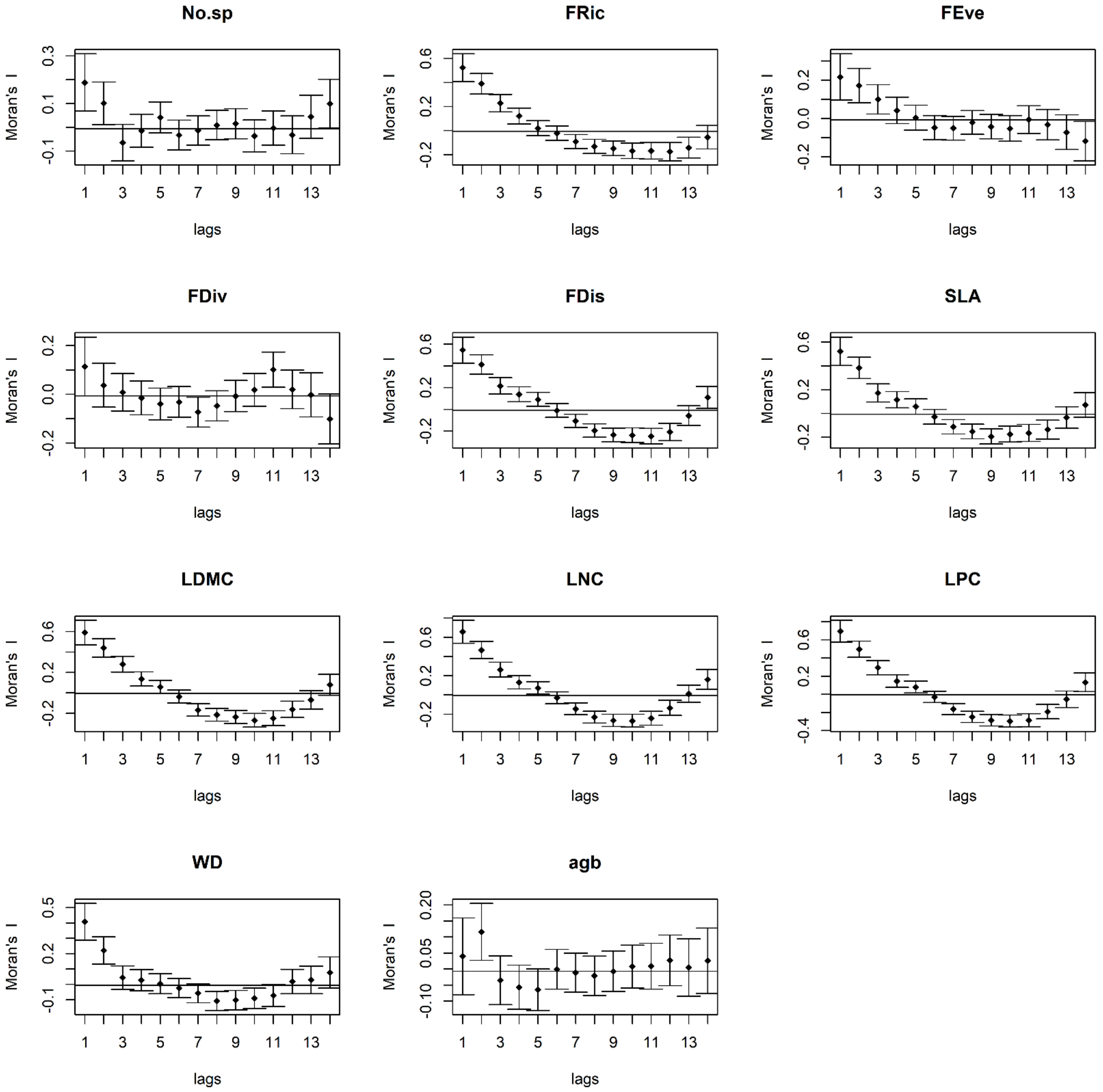

(b) Jianfengling forest dynamics plot

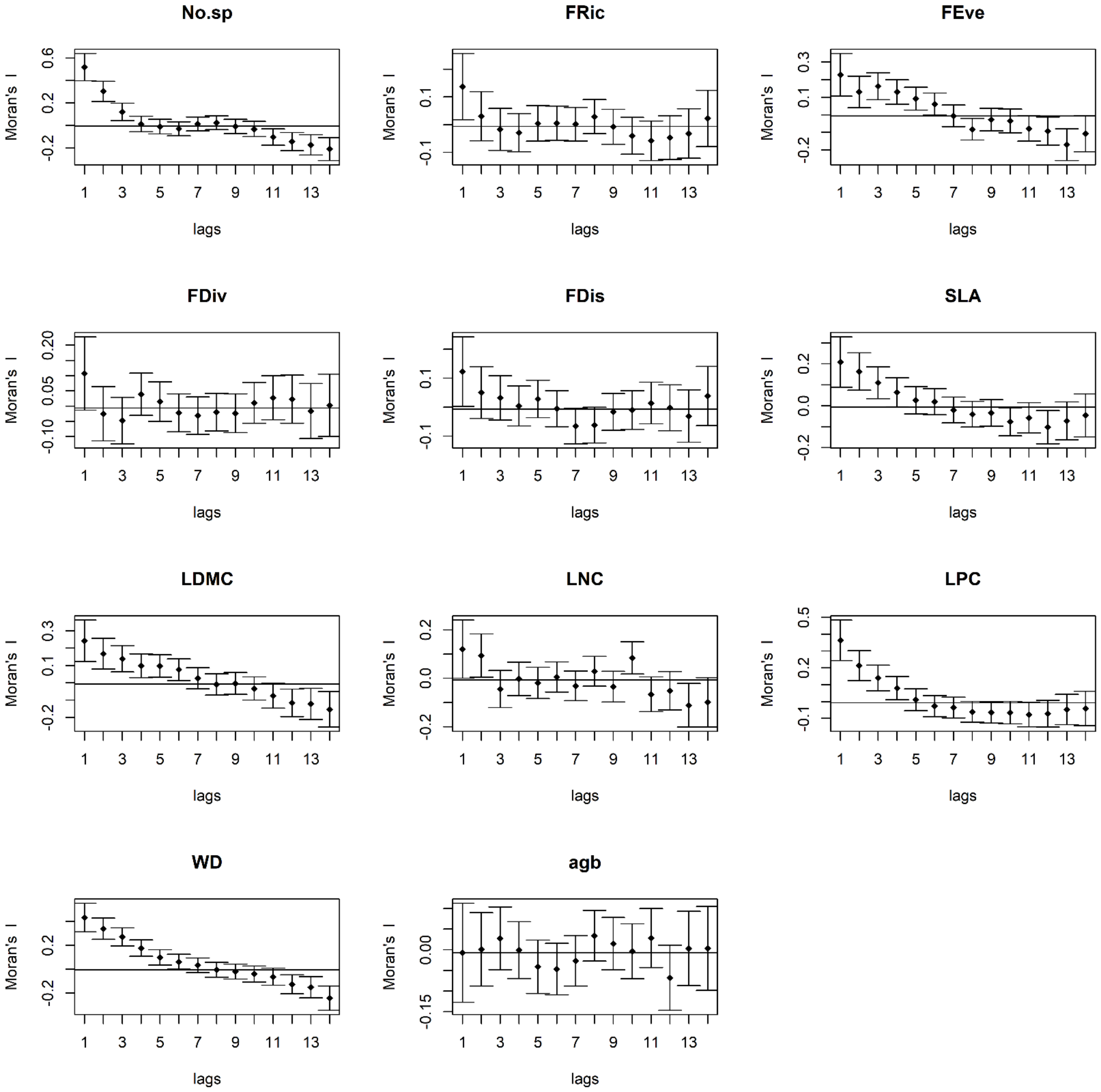

(c) Jiaohe forest dynamics plot

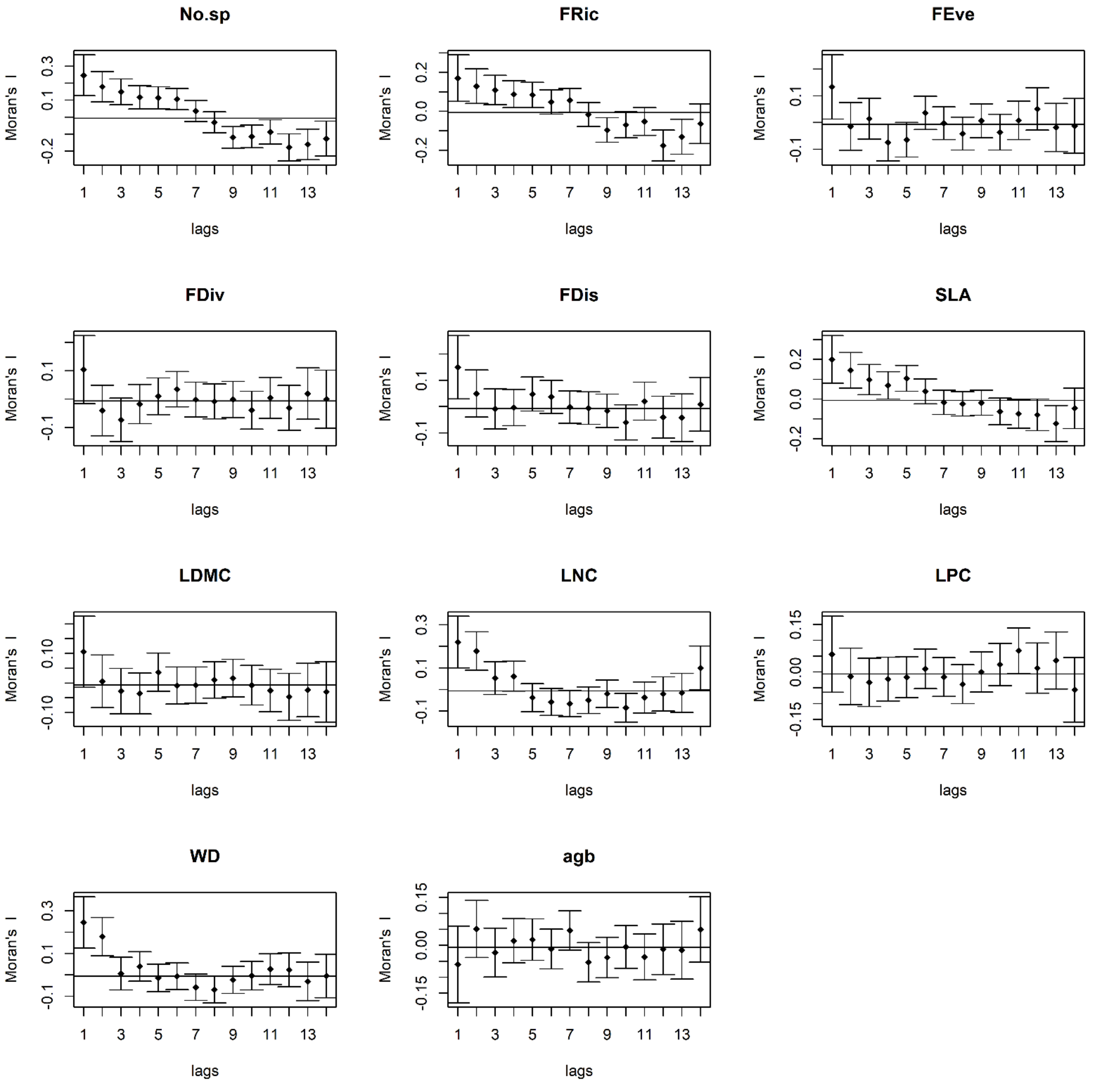

(d) Lushuihe forest dynamics plot

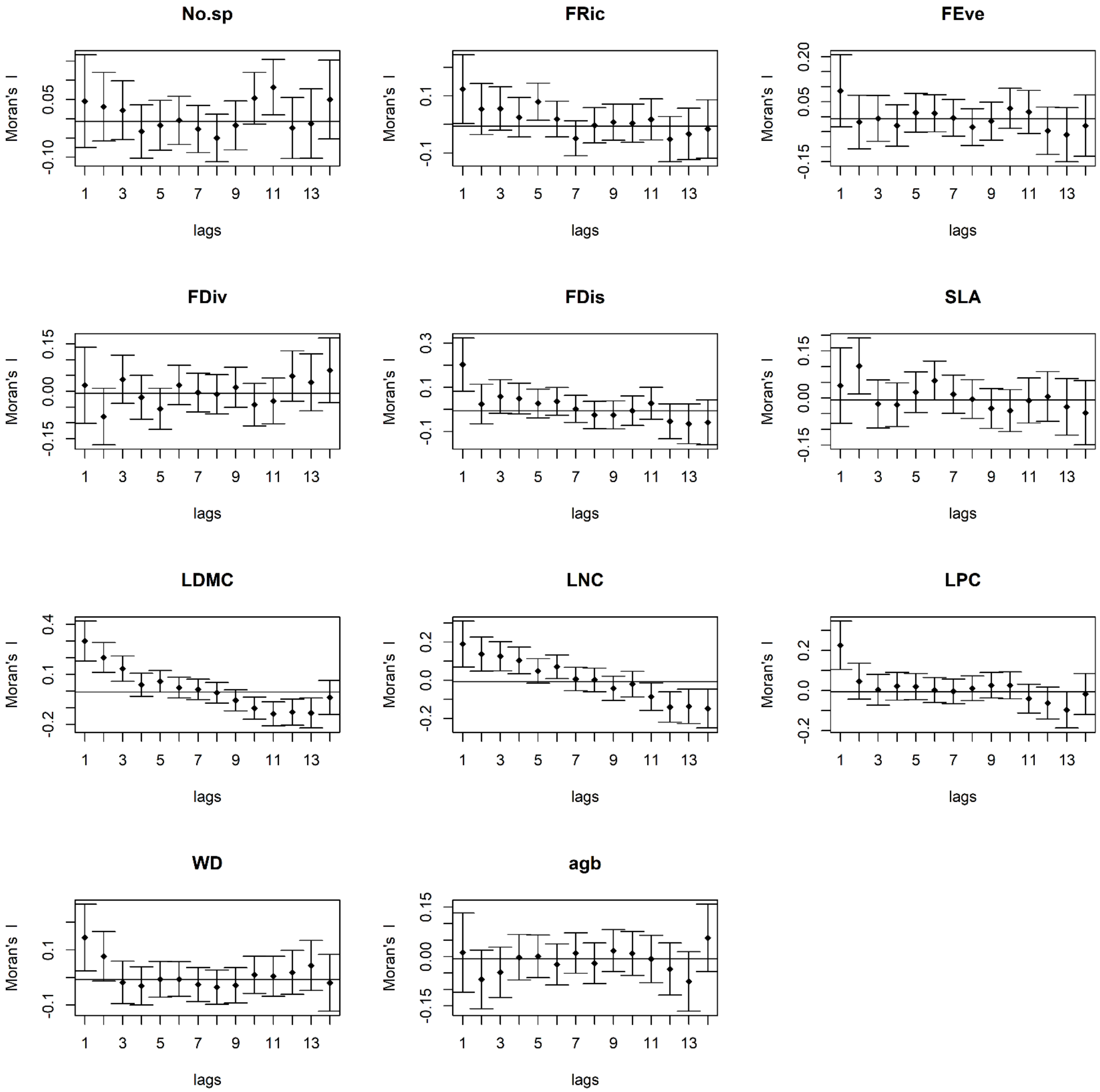

Supplement: Supplementary file 1 — Data S1. Supporting Information. [file ECE3-13-e10487-s001.pdf]
